# Identification of Secondary Metabolites from the Lichen *Hypotrachyna enderythraea* (Zahlbr.) Hale by HPLC-ESI-MS/MS

**DOI:** 10.3390/molecules31060954

**Published:** 2026-03-12

**Authors:** Fernando Carrasco, Wilfredo Hernández, Nino Castro, Nelly Sivipaucar, Bruno Bongiorno, Oscar Chupayo, Cesar Raposo, Lúcia A. Silva, Jesus M. Rodilla, Eduardo Carrasco, Juan Z. Dávalos

**Affiliations:** 1 Facultad de Ingeniería, Universidad de Lima, Av. Javier Prado Este 4600, Lima 15023, Peru; rhenand@ulima.edu.pe; 2 Departamento Académico de Química Orgánica, Escuela de Química, Facultad de Química e Ingeniería Química, Universidad Nacional Mayor de San Marcos, Calle German Amezaga 375, Lima 15081, Peru; ocastrom@unmsm.edu.pe; 3 Facultad de Ciencias Naturales y Matemática, Universidad Nacional Federico Villarreal, Jr. Río Chepen s/n, El Agustino, Lima 15007, Peru; sivipaucarnellyelena@gmail.com (N.S.); brunobongiornoe@gmail.com (B.B.); oscarchupayo@gmail.com (O.C.); 4 Mass Spectrometry Service, NUCLEUS, University of Salamanca, 37008 Salamanca, Spain; raposo@usal.es; 5 Faculdade de Ciencias, Departamento de Quíımica and Fiber Materials and Environmental Technologies (FibEnTech-UBI), Universidade da Beira Interior, R. Marquês de D’Ávila e Bolama, 6201-001 Covilhã, Portugal; mlas@ubi.pt (L.A.S.); rodilla@ubi.pt (J.M.R.); 6 Facultad de Ciencias Física, Universidad Nacional Mayor de San Marcos, Lima 15081, Peru; ecarrascoso@unmsm.edu.pe; 7 Instituto de Química-Física “Blas Cabrera”, CSIC, Serrano 119, 28006 Madrid, Spain; jdavalos@iqf.csic.es

**Keywords:** *Hypotrachyna* species, secondary metabolite profiling, lichen, UHPLC-MS-MS

## Abstract

In this study, sixteen secondary metabolites, including two chromones, four dibenzofurans, three lipids, three depsides, two aromatic compounds, a quinone, and a terpene, were detected in the methanol:acetone (1:1 *v*/*v*) extract of the lichen *Hypotrachyna enderythraea* (Zahlbr.) Hale, using High-Performance Liquid Chromatography coupled to Orbitrap Electrospray Ionization tandem Mass Spectrometry (HPLC-Orbitrap ESI tandem MS/MS). These metabolites were characterized by analysis of their exact molecular masses and corresponding fragmentation patterns. The retention times of the identified metabolites were compared with those of standard compounds, confirming the presence of naturally occurring bioactive compounds. Density Functional Theory (DFT) calculations were employed to investigate preferential deprotonation sites in representative polyprotic metabolites. All these findings may contribute to expanding the spectrum of compounds identified within the genus *Hypotrachyna* and to evaluating their potential biological activities.

## 1. Introduction

Lichens constitute a group of organisms formed by symbiotic associations between a mycobiont and a photobiont [1,2]. These organisms inhabit environments with severe climatic conditions; therefore, several metabolites that are part of their composition are exclusive to lichens [3]. Some of them include usnic acid and pulvinic acid derivatives, which function as highly effective UV-absorbing agents [3]. Approximately 1050 metabolites have been isolated from lichens [3,4], of which about 80% are unique to these organisms [5]. These metabolites include depsides, depsidones, dibenzofurans, anthraquinones, napthoquinones, xanthones, phenols, polysaccharides, lipids and ethers [6,7,8]. Some of these compounds found in lichens exhibited a wide spectrum of pharmacological properties such as antimicrobial, antioxidant, anticancer, antiviral, antidiabetic and antineurodegenerative activities [1,9].

On the other hand, it is known that the *Parmeliaceae* family is the largest family of lichen-forming ascomycetes [10]. According to recent studies, this family comprises 79 genera and 2726 species [11], including the genus *Hypotrachyna*, which consist of more than 260 species [12]. This genus has been reported in America and tropical Asia at an elevation above 1300 m [13]. In Peru, *Hypotrachyna* represents the second most species-rich genus distributed throughout the country [8]. Members of the genus *Hypotrachyna* are foliose lichens characterized by lobed thalli with branched rhizines and lecanorine apothecia, and their identification is carried out by different chemical reactions in specific tests. Ecologically, lichens are typically found in humid montane forests, growing epiphytically on tree bark or occasionally on rocks, where they contribute to nutrient cycling and serve as bioindicators of air quality due to their sensitivity to atmospheric pollutants [14].

High-Performance Liquid Chromatography (HPLC) is a powerful analytical technique used to separate, detect, and quantify analytes in liquid mixtures [15]. Due to its high sensitivity and resolution, HPLC can be coupled with various types of detectors, the most notable being mass spectrometers (MS), such as time of flight (TOF), triple quadrupole, Orbitrap or ion traps [16,17,18]. These spectrometers with fragmentation methods installed are known as tandem MS/MS spectrometers. The most used ionization techniques in MS are electrospray ionization (ESI) and atmospheric chemical ionization (APCI). ESI is a soft ionization technique and a powerful tool for ionizing thermolabile and moderately polar compounds [16,17]. It typically yields even-electron ions, such as protonated molecules [M+H]^+^, metal-adduct cations [M+Metal]^+^ and deprotonated molecules [M−H]^−^. The formation of radical ions (M^•+^) due to electron loss of an electron is less common in ESI [16,19]. Therefore, HPLC coupled with ESI tandem mass spectrometry (HPLC-ESI-MS/MS) is well-suited for metabolite identification in lichen extracts, particularly using ultra-high resolution Mass Spectrometers, such as the Orbitraps [3,7,8,20]. The study of fragmentation patterns in Mass Spectrometry relies on identifying the most probable ionization or deprotonation sites, which are particularly complex in polyprotic metabolites. In this context, computational chemistry—through Density Functional Theory (DFT)—provides substantial support due to its ability to generate consistent results that correlate well with experimental evidence [7,21,22,23].

In this context, the structure–fragmentation relationships of lichen metabolites were investigated via ESI-Qq-TOF-MS/MS in negative mode. Ten compounds including phenols, depsides, depsidones, and dibenzofurans were analyzed, identifying diagnostic fragments from the loss of neutral molecules (CO, CO_2_, CH_3_OH, CH_3_CH_2_OH and CH_2_=CH_2_) and odd-electron ions in sekikaic, lobaric, and usnic acids. A total of fifteen metabolites were characterized in extracts from *Parmotrema grayana* and *Heterodermia obscurata*. These experimental findings were supported by DFT calculations (B3LYP/6-31G) to identify probable deprotonation sites in polyprotic metabolites.

In a recent study, the metabolomic profiles of two methanolic extracts from lichens of the *Parmotrema* genus (*P. andinum*, and *P. robustum*) have previously been reported [8]. This study was conducted using ultrahigh-performance liquid chromatography diode array detection and ESI quadrupole Orbitrap Mass Spectrometry (UHPLC-DAD-ESI-Q-Orbitrap-MS/MS), operating in negative ion mode. A total of 54 compounds were identified in these lichen species, including depsides, depsidones, lipids, aromatic compounds, diphenyl ethers, and dibenzofurans.

Similarly, Kumar et al., 2018 [6] conducted a comparative phytochemical study on five lichen extracts belonging to the genera *Parmotrema*; *P. tinctorum (Delise exNyl.) Hale*, *P. andinum (Mull. Arg.) Hale*, *P. praesorediosum (Nyl.) Hale*, *P. grayanum (Hue) Hale*, and *P. austrosinense* (Zahlbr.) *Hale*. The chemical characterization of the lichen extracts was performed using UPLC-photodiode array detection-quadrupole TOF–MS/MS (UPLC-PDA-QToF-MS/MS) and UPLC-APCI-MS/MS with multiple reaction monitoring (MRM). The results revealed the presence of common secondary metabolites in all five extracts, including orselinic acid, lecanoric acid, methyl β-orselinate, norlobaridone and atranorin.

Parrot et al., 2015 [24] compared the metabolic profiles of nine lichen species belonging to the genera *Lichina*, *Collema* and *Roccella*. The extracts were analyzed using HPLC-DAD-ESI -MS. Nine major compounds were identified as β-orcinol, orselinic acid, choline sulfate, roccelic acid, montagnetol, lecanoric acid, erythrin, lepraric acid and acetylportentol.

There are very few metabolomic studies on lichens of the genus *Hypotrachyna*. The literature reports only two HPLC-MS/MS metabolomic studies on extracts of *Hypotrachyna cirrhata* using acetone [25] and ethyl lactate [26] as solvents. In the acetone extract, 22 metabolites were identified, and some of them were isolated including salazinic acid, atranorin, lecanoric acid and lichesterinic acid [25]. In the ethyl acetate extract, 76 metabolites that included depsides (thamnolic, haemathamnolic and hypotrachynic acids) and depsidones (consalazinic and salazinic acids and their derivatives, as well as menegazziaic, norstictic and psoromic acids) as predominant compounds [26] were detected. The metabolites common to both studies were consalazinic acid, salazinic acid, tamnolic acid, atranorin and usnic acid [25,26].

Additionally, four new β-orcinol metabolites (hypotrachynic acid, deoxystic acid, cryptostictinolide and 8’-methylconstictic) were isolated from *Hypotrachyna revoluta* [27], and two new β-orcinol metabolites (hypotrachynin A and B) were isolated from *Hypotrachyna caraccensis* [28]. More recently, Sepulveda et al. reviewed the literature on metabolites present in *Hypotrachyna* species and reported a wide variety of compounds across twelve species collected from different locations; however, no clear chemotaxonomic patterns were identified [26]. The diversity of metabolites within lichens of the same genus appears to depend largely on environmental and habitat variability [26].

Several lichen extracts from the genus *Hypotrachyna* exhibiting antioxidant activity have been reported [25,27,28]. The methanolic extract of *H. caraccensis* [28] and the acetonic extract of *H. cirrhata* [25] showed 91.695 and 95.79% scavenging activity against the 2,2-diphenyl-1-picrylhydrazyl (DPPH) radical, respectively [25,28]. These results are likely associated with the presence of phenolic compounds, as well as depsides and depsidones, which enhance the antioxidant properties of these extracts [25,27,28].

The aim of this research is to carry out the first systematic and comprehensive metabolomic study of the methanol–acetone extract from the lichen *Hypotrachyna enderythraea* (Zahlbr.) Hale, collected in Arequipa, Peru, using HPLC–Orbitrap-ESI-MS/MS to identify and characterize the secondary metabolites present in this species. Furthermore, Density Functional Theory (DFT) calculations are integrated to validate the experimental results by modeling preferential deprotonation sites, providing a mechanistic rationale for the stability of the observed anions. This research provides a robust framework for the bioprospecting of lichen metabolites with potential biological activities.

## 2. Results and Discussion

**Figure 1:**
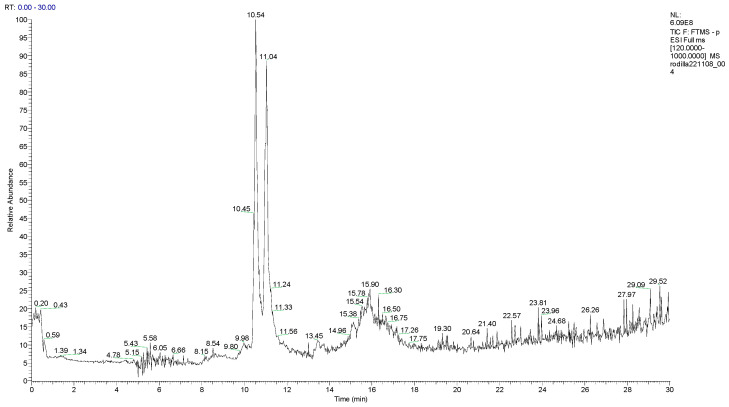
Chromatogram of *Hypotrachyna enderythraea* (Zahlbr.) Hale on negative mode.

In this study, sixteen compounds were detected in the methanol:acetone (1:1, *v*/*v*) extract of the lichen *Hypotrachyna enderythraea* (Zahlbr.) Hale using HPLC–Orbitrap–ESI–MS/MS. Thirteen metabolites were fully identified in negative ionization mode (ESI^−^) and one in positive ionization mode (ESI^+^) (Figure 1 and Figure S1). The results are summarized in Table 1. Two dibenzofurans, namely (R)-usnic acid and isousnic acid, exhibited ionization in both positive and negative modes. The identified compounds comprised two chromones, four dibenzofurans, three lipids, three depsides, two aromatic compounds, one quinone, and one terpene.

### 2.1. HPLC-Orbitrap ESI-MS/MS Analysis

***Chromones***

**Figure 2:**
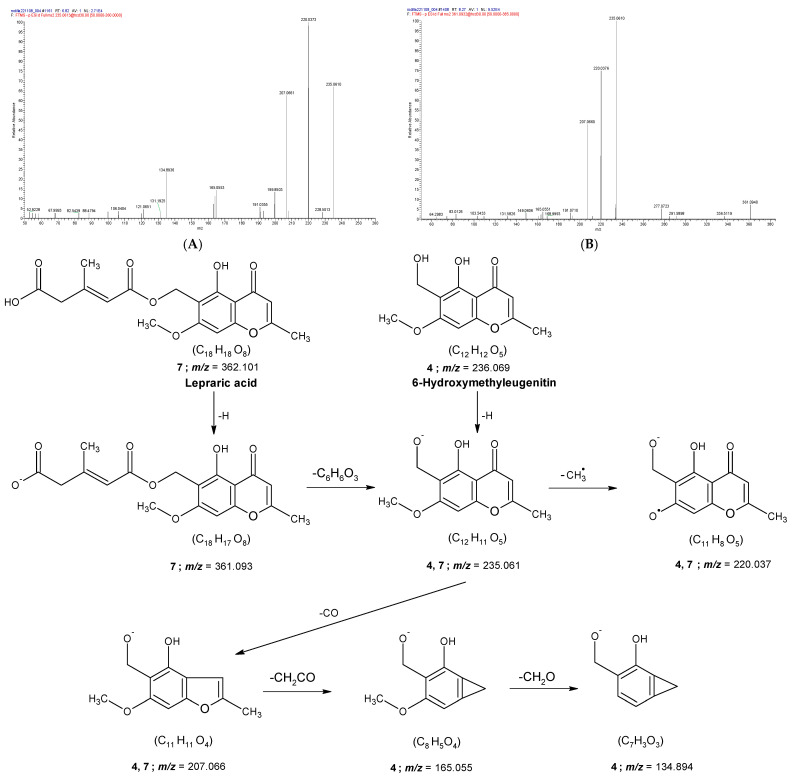
MS^2^ spectra of the [M-H]^−^ anions corresponding to (**A**) 6-hydroxymethyleugenitin (4) and (**B**) lepraric acid (**7**). The proposed fragmentation pathways for compounds **4** and **7** are shown below.

Two chromones (peaks **4** and **7**) were identified by HPLC-Orbitrap-ESI-MS/MS analysis (see Table 1 and Figure 2). Peaks **4** and **7** correspond to deprotonated molecular ions [M−H]^−^ at *m/z* 235.0614 and 361.0934, respectively [29].

For peak **7**, the loss of a C_6_H_6_O_3_ fragment from the precursor ion [M−H]^−^ can occur through displacement reactions [16], in which the negative charge is transferred to the fragment ion [M–H–C_6_H_6_O_3_]^−^ (*m/z* 235.0614). This *m/z* value is identical to that observed for ionized 6-hydroxymethyleugenitin (peak **4**, [M–H]^−^). The MS/MS spectra of both compounds revealed radical anions at *m/z* 220.037, corresponding to radical ions [M–H–CH_3_]^•−^ and [M–H–C_6_H_6_O_3_–CH_3_]^•−^, respectively, formed by elimination of a methyl radical from the product ions at *m/z* 235.0614 [30,31].

Additionally, fragment ions corresponding to [M–H–CO]^−^ (peak **4**) and [M–H–C_6_H_6_O_3_–CO]^−^ (peak **7**) were detected at *m/z* 207.066, arising from CO elimination associated with chromone ring contraction [31]. This elimination pathway is commonly observed in benzofurans [31], chalcones [32], and quinones [16]. The [M–H–CO]^−^ ion corresponding to peak **7** may undergo further cleavage across the benzofuran furan ring, yielding a fragment at *m/z* 165.055 through radical fragmentation accompanied by the loss of a neutral ketene molecule. This fragmentation mechanism was initially proposed by Givens et al. for radical cations generated by electron impact ionization (EI) [33]. Furthermore, Dias et al. reported the formation of benzocyclopropenium cations via CO loss from the benzofuran ring induced by ESI conditions [34].

Although radical ions generated by electron loss and radical-driven fragmentations are uncommon in ESI, several studies support this fragmentation behavior [7,16,30,31,34]. Finally, the formation of the ion at *m/z* 134.894 involves the elimination of formaldehyde through hydrogen rearrangement [34].

Figure 2 shows the proposed fragmentation pathways for 6-hydroxymethyleugenitin and lepraric acid, which were previously identified in *Roccella fuciformis* (*Roccella* lichens) [35,36,37] and *Ramalina sinensis* [29].

***Dibenzofurans***

Four dibenzofurans corresponding to the peaks **10**, **11**, **12** and **15** were identified (Table 1). Peak **10** (Figure S5), exhibiting an [M–H]^−^ ion at *m/z* 375.1088, was identified as pseudoplacodiolic acid [38,39]. This compound is an isomethoxide derivative of usnic acid with a *trans* ring junction [40]. The fragment ion at *m/z* 299.0399 can be explained by the parallel loss of a methyl group and a methanol molecule, together with decarboxylation of the deprotonated molecular ion [M–H]^−^ [40].

Usnic acid [7,26,40] and isousnic acid [38,41] exhibited identical [M–H]^−^ ions at *m/z* 343.0824 but different retention times (10.6 and 11.2 min, respectively; Table 1). These compounds represented peaks **11** and **12** (Figures S6 and S7) and were considered as structural isomers differing in the substitution pattern of ring A and displaying similar fragmentation pathways. The major product ions for both compounds were [M–H–CH_3_]^•−^ at *m/z* 328.059 and [M–H–C_4_H_3_O_2_–CO]^−^ at *m/z* 231.066. According to the literature, these fragmentations arise from the loss of a methyl radical and retro-Diels–Alder reactions occurring in radical cations (M•^+^) and deprotonated anions ([M–H]^−^), using chemical ionization [42] and ESI techniques [7,40,43,44]. In addition, both compounds generated a radical ion at *m/z* 83.013, formed through an aromatic elimination process [16]. This fragment is relatively stable due to resonance stabilization arising from alternating unsaturations [44].

Kutney et al. extensively investigated the fragmentation patterns of usnic acid and related derivatives, including isousnic acid, usnic acid monoacetate, dihydrousnic acid, usnic acid ethoxide, ethyl acetusnetate, usnetol, deacylusnic acid derivatives, 2-desacetylusnic acid, enaminousnic acid, and usnic acid diacetate, using Chemical Ionization Mass Spectrometry [42].

Usnic acid is one of the most extensively studied lichen metabolites and exhibits a wide range of biological activities, including anti-inflammatory, antiviral, antimicrobial, anticancer, antiprotozoal, antiproliferative, and analgesic effects [45]. This compound has been previously reported in lichens of the genus *Hypotrachyna*, such as *H. cirrhata*, *H. chicitae*, *H. caraccensis*, and *H. hypoalectorialica* [26]. Isousnic acid has also been identified in the lichen *Rhyzoplaca chrysoleuca* [38].

Peak **15** was assigned to 9-methyl-8-*O*-methylpannarate [46], exhibiting an [M–H]^−^ ion at *m/z* 343.0589. The radical fragment ions at *m/z* 328.0589 ([M–H–CH_3_]^•−^) and *m/z* 313.0358 ([M–H–2CH_3_]^••−^) (Figure S10) were formed via sequential loss of methyl radicals.

***Lipids***

Lichen-derived lipids are highly diverse, and they are of great importance in explaining the adaptive capacity of lichens, which are exposed to adverse climatic conditions such as extreme variations related to changes and altitude, high light exposure and salinity levels, and extreme pH levels [47]. Lipids are commonly identified via HPLC-MS/MS analyses. In recent research, several polyhydroxylated lipids were obtained from the ethanolic extract of the lichen *Himantormia lugubris* [48] and the ethyl lactate and the methanolic extracts of the lichens *Hypotrachyna cirrata* [26], *Parmotrema robustum* and *P. andinum* [8] using HPLC-MS/MS.

In the present study, three lipids corresponding to peaks **1**, **13**, and **14** were identified. Peak **1** (Figure S2) was assigned to azelaic acid [49], exhibiting an [M–H]^−^ ion at *m/z* 187.097. Peaks **13** and **14** (Figures S8 and S9) were identified as palmitic acid (C_16_H_32_O_2_) and stearic acid (C_18_H_36_O_2_), with deprotonated molecular ions observed at *m/z* 255.2332 and 283.2646, respectively [50].

***Depsidones***

Depsidones are a class of polyphenolic polyketide compounds biosynthesized via the acetyl–malonate pathway [51]. These metabolites are commonly found in fungi and lichens and exhibit a wide range of biological activities, including antimalarial, antimicrobial, anti-inflammatory, anti-*Helicobacter pylori*, antituberculosis effects, and acetylcholinesterase inhibition [52].

**Figure 3:**
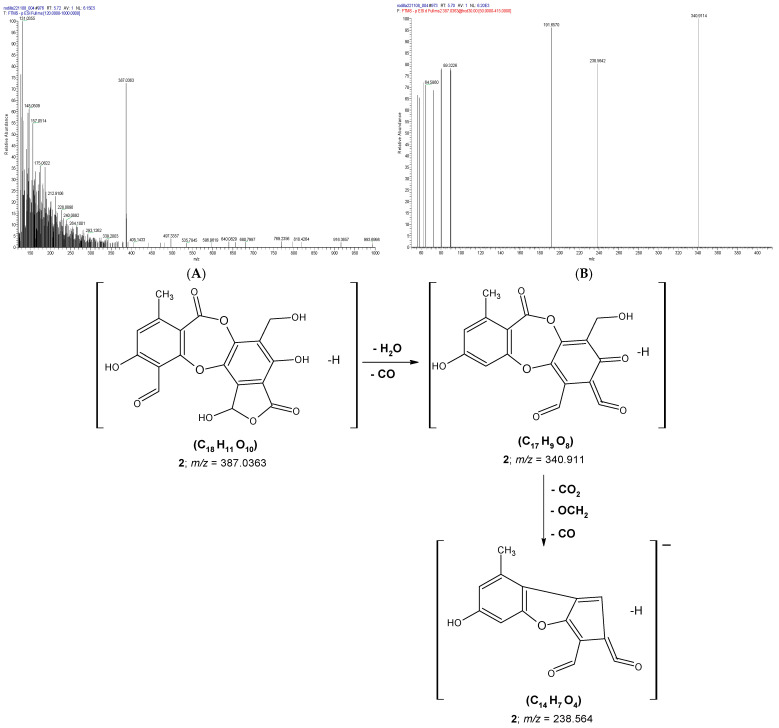
(**A**) MS^2^ spectrum of the [M-H]^−^ anion at *m/z* 387.0363 assigned to salazinic acid (**2**); (**B**) MS^2^ spectrum of fragmentation products of **2**, with the proposed fragmentation pathway shown below.

According to the present results, three depsidones corresponding to peaks **2**, **5**, and **9** were identified (Table 1). Peak **2** (Figure 3) was identified as salazinic acid, exhibiting an [M–H]^−^ ion at *m/z* 387.0363. Fragmentation produced an ion at *m/z* 340.911 ([M–H–H_2_O–CO]^−^) via sequential loss of H_2_O and CO [6,43]. The product ion at *m/z* 238.564 originated from the precursor at *m/z* 340.911 through successive losses of CO_2_, OCH_2_, and CO (Figure 3). The elimination of H_2_O, CO, and CO_2_ is characteristic of depsidone fragmentation patterns [6,51]. Salazinic acid has previously been reported in *H. cirrhata*, *H. quaesita* [26], *P. andinum* [8], *P. tinctorum* [6], *Usnea barbata*, *U. rubicunda*, *U. subfloridana* [53], and *Ramalina var. Crassa* [54].

**Figure 4:**
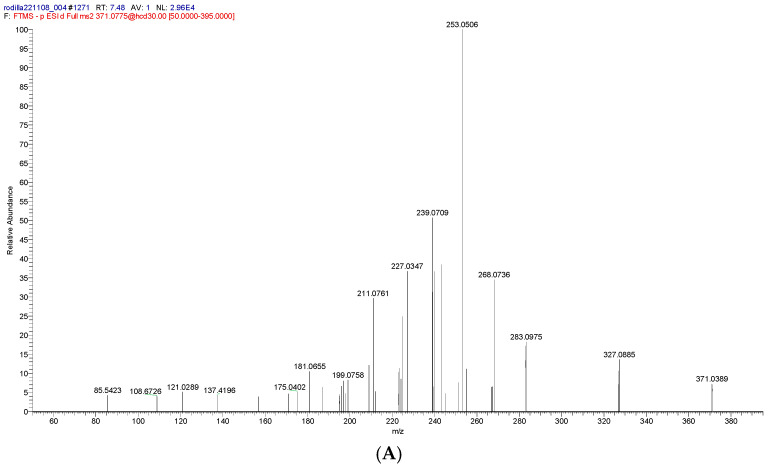

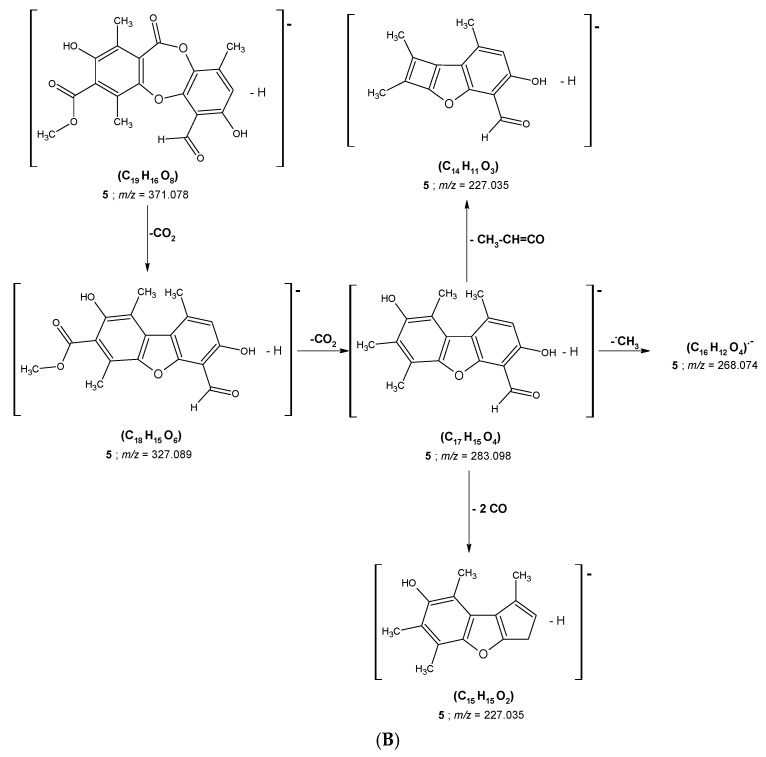
(**A**) MS^2^ spectrum of the [M-H]^−^ anion at *m/z* 371.078 assigned to methyl virensate (**5**); (**B**) proposed fragmentation pathway of **5**.

Peak **5** (Figure 4), assigned to [M–H]^−^ ion at *m/z* 371.078, was identified as methyl virensate. This compound undergoes decarboxylation to yield a characteristic [M–H–CO_2_]^−^ ion at *m/z* 327.089. Further loss of CO_2_ from this ion produces a fragment at *m/z* 283.098, which likely involves migration of the methyl group to the aromatic carbon, facilitating CO_2_ elimination. This rearrangement mechanism in unsaturated esters was previously proposed by Bowie and Williams based on EI mass spectra [55]. The ion at *m/z* 283.098 ([M–H–2CO_2_]^−^) may further lose a methyl radical (–CH_3_^•^) to yield a radical anion at *m/z* 268.074 ([M–H–2CO_2_–CH_3_]^•−^) (Figure 4). Loss of methyl radicals has also been reported for dibenzofuran derivatives bearing methyl substituents on the furan ring [42].

A product ion found at *m/z* 227.035 was also assigned to peak 5. This ion was formed by sequential loss of 2CO_2_ and 2CO units from the pseudomolecular ion (*m/z* 371.078). This obtained fragment ([M–H–2CO_2_–2CO]^−^) has previously been reported for depsidone isomers of methyl virensate [56]. Alternatively, this ion may arise from the sequential loss of 2CO_2_ and CH_3_–CH=CO groups, a fragmentation pattern previously described for norstictic acid [54]. Methyl virensate has been identified in *Pseudocyphellaria physciospora* and *P. granulata* lichens [57]; to the best of our knowledge, this is the first report of methyl virensate in the genus *Hypotrachyna*.

Peak **9** was identified as lobaric acid, exhibiting an [M–H]^−^ pseudomolecular ion at *m/z* 455.172 (Figure S4). This ion may undergo double decarboxylation to produce a fragment at *m/z* 367.191 (C_23_H_27_O_4_). Lobaric acid has previously been identified in lichens of the genera *Parmotrema* [6,8], as well as in members of the Parmeliaceae [53] and Cladoniaceae families [26].

***Aromatic compounds***

**Figure 5:**
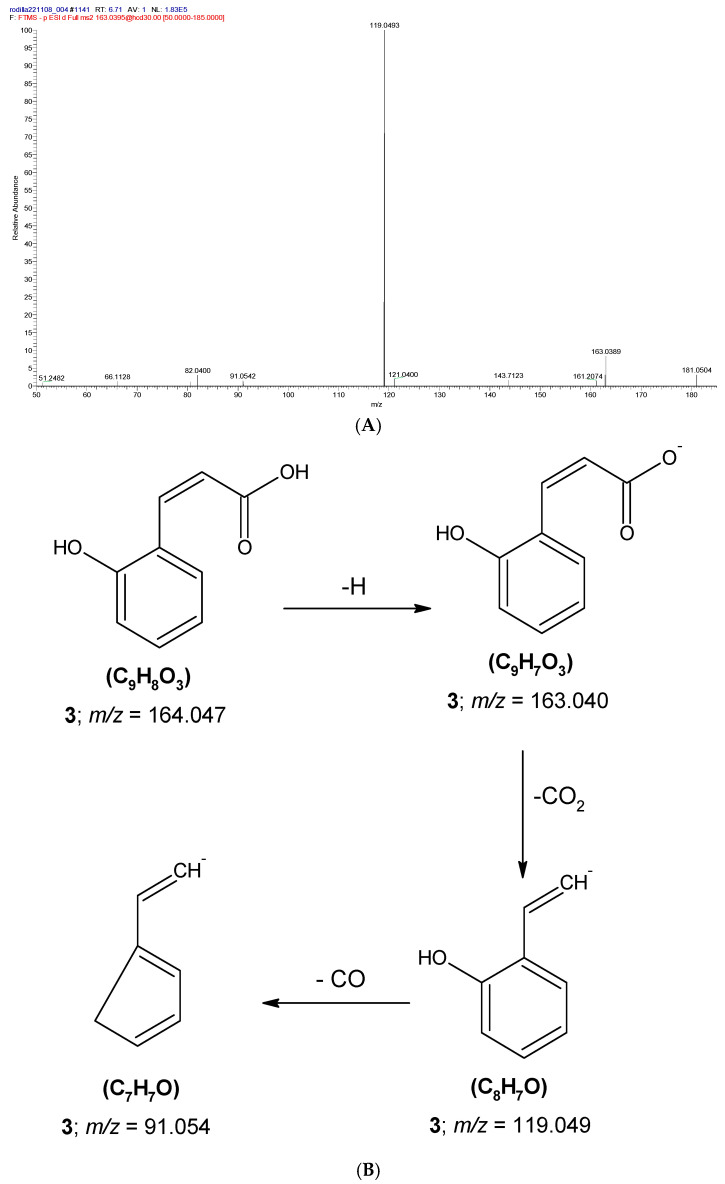
(**A**) MS^2^ spectra of the [M-H]^−^ anion assigned to coumarinic acid (**3**), (**B**) proposed fragmentation pathway of **3**.

Two compounds containing phenyl rings were identified and assigned to peaks **3** and **6** (Table 1). Peak **3** was identified as coumarinic acid. Figure 5 shows the proposed fragmentation of peak **3** in the negative ionization mode. The pseudomolecular ion was detected as [M–H]^−^ at *m/z* 163.040, which underwent decarboxylation with loss of CO_2_ to produce the fragment [M–H–CO_2_]^−^ at *m/z* 119.049. This ion was the most abundant fragment and may be associated with stabilization by the hydroxyl group present in the product ion [31]. Furthermore, the fragment [M–H–CO_2_–CO]^−^ at *m/z* 91.054 was generated from the ion [M–H–CO_2_]^−^ (*m/z* 119.049) through subsequent CO elimination. This fragmentation behavior is characteristic of phenolic acids analyzed by ESI [58]. Coumarinic acid has recently been reported in the lichen *Usnea barbata* [59].

Peak **6** was identified as ethyl haematommate, exhibiting a deprotonated molecular ion [M–H]^−^ at *m/z* 223.061 and product ions at *m/z* 207.029, 181.050, 163.039, and 83.013 (Figure S3). The fragment ion at *m/z* 181.050 is likely formed by loss of a ketene moiety (CH_2_CO) from the pseudomolecular ion. Ethyl haematommate was recently isolated from the ethyl acetate extract of *Stereocaulon graminosum*, and its structure was confirmed by spectroscopic methods and single-crystal XRD [60]. This compound is considered an intermediate in depside biosynthesis [60].

***Antraquinone***

Anthraquinones are natural pigments that have been isolated from plants, lichens, and fungi [61]. Lichens are known to produce unique anthraquinone derivatives that have not been found in higher plants [61,62]. Manojlović et al. isolated six anthraquinone compounds—erythroglaucine, xanthorin, physcion, fallacinal, teloschistin, and emodin—from three *Xanthoria* species. All the isolated anthraquinones exhibited antibacterial activity against different *Pseudomonas* strains [62].

**Figure 6:**
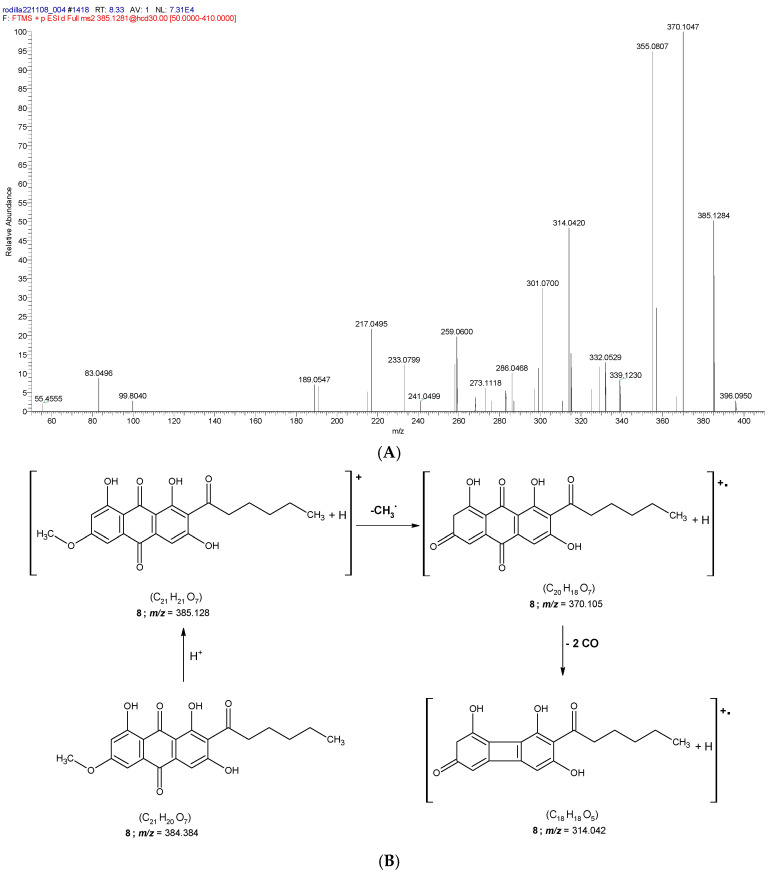
(**A**) MS^2^ spectra of the [M+H]^+^ cation assigned to solorinic acid (8); (**B**) proposed fragmentation pathway of **8**.

According to the present results, peak **8** (Table 1) was identified as solorinic acid (C_21_H_20_O_7_), exhibiting a protonated molecular ion [M+H]^+^ at *m/z* 385.1281. This compound is a pigment originally identified in the lichen *Solorina crocea* [63]. Solorinic acid generated a product ion at *m/z* 370.105 through the loss of a methyl radical. The radical cation at *m/z* 314.042 ([M+H–CH_3_]^•+^) was formed via successive losses of two CO units from the product ion observed at *m/z* 370.105. The formation of radical cations among product ions is a common feature in the fragmentation patterns of anthraquinones analyzed in positive ionization mode [64] (see Figure 6).

***Terpenes***

One terpene corresponding to peak **16** was identified as portentol (C_17_H_26_O_5_), exhibiting anion [M-H]^−^ at *m/z* 309.174 (Figure S11). Portentol was originally identified in lichens of the genus *Roccella portentosa* [37].

Usnic and salazinic acids have been identified in other species of the genus *Hypotrachyna*. Usnic acid has been identified in *H. cirrhata*, *H. chicitae*, *H. caraccensis* and *H. hypoalectorialica,* whereas salazinic acid has been identified in *H. cirrhata* and *H. quaesita* [26].

The evaluation of lichen extracts in both positive and negative ionization modes provides complementary information for metabolomic characterization. However, Olivier-Jimenez et al. (2019) [65] reported that the occurrence of acidic functions within many lichen metabolites strongly favors their analysis in negative-ion mode, consistently with former reports. This explains why the literature predominantly employs ESI^−^ for lichen metabolomics, since deprotonation of acidic compounds gives stable anions with enhanced detection sensitivity. In our study, the negative-ion chromatogram revealed the highest number of metabolites, confirming the suitability of this approach. Nevertheless, the inclusion of positive-ion mode remains valuable for broadening chemical coverage, particularly for neutral or basic metabolites that ionize preferentially under protonation conditions.

**Table 1:** Identification of metabolites in the methanol:acetone (1:1, *v*/*v*) extract of the lichen *H. enderythraea* (Zahlbr.) Hale by HPLC-Orbitrap ESI-MS/MS.

Peak	Compounds	[M−H]	Ret. Time (min)	Theoretical (m/z)	Experimental (m/z)	Accuracy (ppm)	Fragmentation Pattern	Type	Ref.
**1**	Azelaic acid	C_9_H_15_O_4_	4.23	187.097	187.097	0.0	169.0864; 125.0962; 123.0808; 97.0649	L	[49]
**2**	Salazinic acid	C_18_H_11_O_10_	5.72	387.0361	387.036	−0.26	238.5642; 191.657; 89.3226; 79.958	D	[6,43,51]
**3**	Coumarinic acid	C_9_H_8_O_3_	6.7	163.0394	163.04	3.68	119.0493; 91.0542	A	[59]
**4**	6-Hydroxymethyleugenitin	C_12_H_12_O_5_	6.82	235.0612	235.061	−0.85	220.0373; 207.0661; 165.0553; 134.8936	C	[29]
**5**	Methyl virensate	C_19_H_16_O_8_	7.49	371.0774	371.078	1.62	268.0736; 253.0506; 239.070; 227.0347	D	[57]
**6**	Ethyl haematommate	C_11_H_12_O_5_	7.56	223.0609	223.061	0.45	207.0297; 181.05; 163.0391; 83.0127	A	[60]
**7**	Lepraric acid	C_18_H_18_O_8_	8.26	361.0932	361.093	−0.55	235.061; 227.0723; 220.0376; 207.066	C	[29,35,36,37]
**8**	Solorinic acid *	C_21_H_21_O_7_	8.34	385.1293	385.128	−3.4	370.1047; 355.0807; 314.042; 301.07	AQ	[63]
**9**	Lobaric acid	C_25_H_27_O_8_	9.24	455.1714	455.172	1.32	367.1911; 309.1143; 295.0997; 281.0828	D	[6,8,26,53]
**10**	Pseudoplacodiolic acid	C_19_H_19_O_8_	9.69	375.1086	375.109	1.07	299.0393; 255.0666; 231.0663; 83.0127	DBF	[38,39]
**11**	(R)-Usnic acid **	C_18_H_15_O_7_	10.6	343.0808	343.082	3.5	328.0594; 259.0616; 231.0663; 83.0128	DBF	[7,26,40]
**12**	Isousnic acid **	C_18_H_14_O_7_	11.04	343.0808	343.082	3.5	328.0592; 313.0356; 231.0663; 83.0128	DBF	[38,43]
**13**	Palmitic acid	C_16_H_31_O_2_	13.47	255.233	255.233	0.0	177.689; 116.9272; 97.5035	L	[50]
**14**	Stearic acid	C_18_H_35_O_2_	15.18	283.2644	283.265	2.12	259.6608; 200.7311; 138.0842; 86.0265	L	[50]
**15**	9-Methyl 8-O-methylpannarate	C_18_H_15_O_7_	15.2	343.0825	343.083	1.46	328.0589; 313.0358; 231.0659; 83.0127	DBF	[46]
**16**	Portentol	C_17_H_25_O_5_	21.47	309.1742	309.174	−0.65	235.8537; 122.9746; 96.959; 79.9564	P	[37]

A aromatic, D depsidone, DBF dibenzofuran, L lipid, C chromone, AQ anthraquinone, P polyketide. * Solorinic acid was measured in positive ion mode (ESI^+^). ** These analytes were also measured in positive ion mode (ESI^+^) (Table S1).

### 2.2. Computational Studies

The prediction of deprotonation sites in selected polyprotic metabolites was carried out using Density Functional Theory (DFT) calculations. Molecular geometries were optimized with the Gaussian 16 package, and conformational searches were performed to identify the lowest-energy structures. For each optimized structure, the absolute electronic energy (EE) was determined to confirm the absence of imaginary frequencies. Thermal corrections at 298 K were applied to compute the enthalpy values (*H*). The neutral molecule was identified as the global minimum and compared with its corresponding anion (deprotonated molecule) by applying the acidity parameters in the gas phase, that is, changes in Gibbs energy and in the enthalpy of the deprotonation reaction [21] (see Table 2).

**Figure 7:**
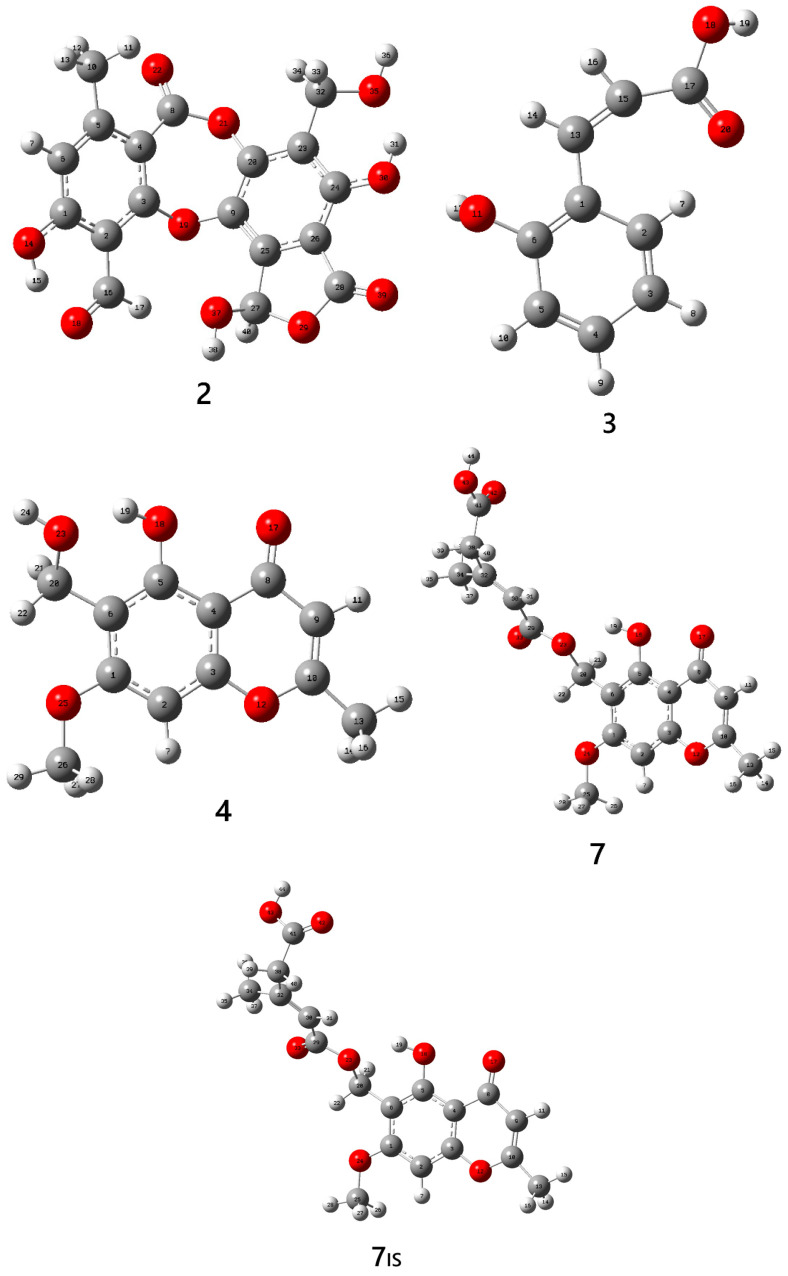
Molecular geometry of salazinic acid (**2**), coumarinic acid (**3**), 6-Hydroxymethyl-eugenitin (**4**) and lepraric acid (**7** and **7**_IS_) optimized at B3LYP/6-311++G(d,p) level of theory.

For salazinic acid (**2**), four hydroxyl sites (14-O, 30-O, 35-O, and 37-O) were evaluated as potential deprotonation sites (Figure 7). Among these, the hydroxylated oxygens at sites 30-O and 35-O have the lowest and identical deprotonation enthalpies (∆*H*_acid_ = 313 kcal/mol), reflecting the greater stability of the deprotonated anions and indicating that these are the most favorable sites for deprotonation, or the “more acidic” sites, which correspond to the lowest *GA* (=305.7 kcal/mol) value. On the contrary, 14-O and 37-O are the least favorable sites, with greater values for the acidity parameters *GA* = 317.7 and 320.3 kcal/mol (∆*H*_acid_ = 326.2 and 328.8 kcal/mol), respectively.

For coumarinic acid (**3**), two possible deprotonation sites were identified: carboxyl group at the 18-O site and the hydroxyl group at the 11-O site (Figure 7). Deprotonation at 11-O was found to be more favorable (*GA* = 322.7 kcal/mol) that at the 18-O site (*GA* = 330.5 kcal/mol). These results are in good agreement with those found by Guerrero et al. [22], who demonstrated that the hydroxyl group of coumaric acid, in the gas phase, is more acidic than the carboxyl group. In addition, Musharraf et al. [7] recently showed that the carboxylate site at C-70 in lobaric acid is the most favorable to carry out deprotonation compared to the hydroxyl site at C-20. These metabolites were identified from the lichens *Parmotrema grayana* and *Heterodermia obscurata*.

With respect to compound 6-Hydroxymethyleugenitin (**4**), the two possible deprotonation sites (hydroxyl groups at 23-O and 18-O) exhibited the same *H* and *G* values.

In the case of lepraric acid (**7**), two neutral conformers (**7** and **7**_IS_) were identified with the same thermodynamical stability (∆*G* and ∆*H* < 1 kcal/mol). Deprotonation of 43-O site, corresponding to the carboxyl group, was practically the same as the deprotonation at the 18-O site, associated with the hydroxyl group. The differences in *GA* (or ∆*H*_acid_) between both sites were less than 0.5 kcal/mol. These results show that the deprotonation of polyprotic metabolites can take place at hydroxyl or carboxyl groups, which would stabilize the negative charge distribution in the anions after deprotonation process. This similarity in deprotonation energies between two possible sites has also been observed in functional groups of the same type (–OH) but located at different positions, e.g., methyl-β-orcinolcarboxylate, where both hydroxyl groups exhibited comparable deprotonation energies [7].

## 3. Materials and Methods

### 3.1. Materials

All solvents and reagents employed in this study were of analytical grade, purchased from Sigma-Aldrich (St. Louis, MO, USA) or Merck (Darmstadt, Germany), and were used without further purification. The chemical materials included methanol, acetone, PTFE membrane filters 0.45 μm, usnic acid, gyrophoric acid, caffeine, buspirone hydrochloride, n-butylamine, sodium dodecyl sulfate, and taurocholic acid. All reagents used correspond to certified analytical standards, ensuring both the reproducibility and the traceability of the analyses.

### 3.2. Methods

#### 3.2.1. Lichen Collection and Identification

**Figure 8:**
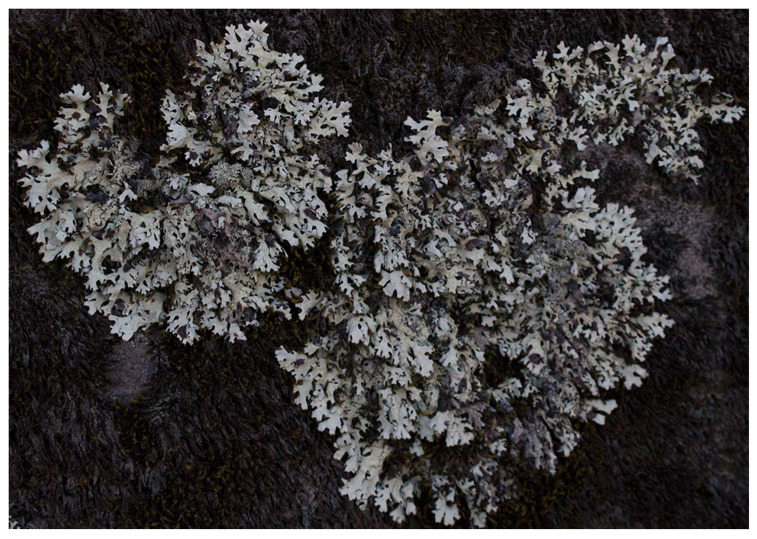
*Hypotrachyna enderythraea* (Zahlbr.) Hale.

*H. enderythraea* (Zahlbr.) Hale (50 g) (Figure 8) was collected in April of 2022 from Arequipa, Peru, S16°28′41.4″ S 71°19′08.5″ W; 3356 m.a.s.l). A voucher specimen (N° 009-2022) was deposited in the herbarium of the “Instituto Científico Michael Owen Dillon”. The sample was identified by the lichenologist Daniel Ramos Aranibar.

#### 3.2.2. Preparation of the Extract

The entire thallus of *H. enderythraea* was manually cleaned to remove solid impurities and then placed in a paper box and dried at room temperature to avoid direct exposure to sunlight. The paper box was changed daily until the lichen was completely dried (3 days) [66]. We used 10 g of the processed lichen to extract it three times with a methanol:acetone (1:1 *v*/*v*) mixture (100 mL used for each extraction, during 24 h per extraction). The extracts were filtered, combined and then concentrated in a rotary vacuum evaporator at 40 °C to obtain the final extract (80 mg).

#### 3.2.3. HPLC Orbitrap-ESI-Tandem MS/MS

High-Performance Liquid Chromatography (HPLC) coupled with Orbitrap Electrospray Ionization tandem Mass Spectrometry, HPLC–Orbitrap ESI–MS/MS (Mass Spectrometry Service-NUCLEUS, University of Salamanca, Salamanca, Spain), was used to determine the profile of secondary metabolites present in the extract of the lichen *Hypotrachyna enderythraea (Zahlbr.)*. For the analysis, 2 mg of the extract was dissolved in 2 mL of methanol and filtered through PTFE membrane filters (0.45 μm pore size). For each experiment, 10 µL of the filtrate was injected into the HPLC-Orbitrap ESI-MS/MS system [2].

**HPLC**

A Thermo Fisher Vanquish HPLC system equipped with a binary pump (Model H; Thermo Fisher Scientific, Bremen, Germany) and an autosampler (Split Sampler HT; Thermo Fisher Scientific, Bremen, Germany), controlled by Xcalibur 2.3 software, was used. Chromatographic separation was achieved using a Kinetex XB-C18 column (Phenomenex; 100 mm × 2.1 mm, 2.6 µm particle size). The mobile phase consisted of 0.1% formic acid in water (A) and acetonitrile (B). The gradient program was as follows (time [min], %A): (0.0, 50); (20.0, 0); (25.0, 0); (26.0, 50). The flow rate was set at 0.2 mL/min, and the injection volume was 10 µL. The total analysis time was 30 min [2]. Xcalibur 2.3 (Thermo Fisher Scientific, Bremen, Germany) and TraceFinder 3.2 (Thermo Fisher Scientific, San José, CA, USA) software were used for HPLC control and data processing, respectively. Usnic acid and gyrophoric acid were used as reference standards.

**Orbitrap ESI tandem-MS/MS**

A Thermo Orbitrap QExactive Focus mass spectrometer (Thermo Fisher Scientific, Bremen, Germany), equipped with an electrospray ionization (ESI) source operating in both positive and negative ion modes, was used. Mass calibration was performed in positive and negative modes using caffeine and *n*-butylamine (Sigma-Aldrich, USA), respectively. Taurocholic acid, buspirone hydrochloride, and sodium dodecyl sulfate (Sigma-Aldrich, Saint Louis, MO, USA) were also used for mass calibration. The optimized instrumental parameters were as follows: ESI operated in negative and positive ion modes; spray voltage −3.8 kV; sheath gas 30; auxiliary gas 10; auxiliary gas heater temperature 310 °C; capillary temperature 320 °C; and S-lens RF level 55. For compound confirmation, targeted MS/MS analyses were performed using an inclusion list with a 30 s time window. The Orbitrap analyzer operated in both positive and negative modes at a resolution of 17,500 FWHM (*m/z* 200). Mass spectra were acquired over the *m/z* range of 100–1000, using a normalized collision energy of 30. The Auto MS^2^ mode was applied for data reprocessing. Full-scan resolution was set to 30,000, and the mass accuracy error was maintained below 1.5 ppm [2].

### 3.3. Computational Approaches

The molecular structures of compounds **2**, **3**, **4**, and **7** (Figure 7) were optimized in the gas phase using Density Functional Theory (DFT) with the Becke three-parameter hybrid functional combined with the Lee–Yang–Parr correlation functional (B3LYP) [67], in conjunction with the 6-311++G(d,p) basis set [68], as implemented in the Gaussian 09 package [66]. Geometry optimizations were performed without symmetry constraints, and harmonic vibrational frequency calculations were carried out at the same level of theory to confirm the nature of the stationary points [69]. The deprotonated sites were identified through structural analysis and thermodynamic profiles (ΔH, ΔG), enabling a comprehensive evaluation of competing ionization pathways. These specific compounds were selected because they possess multiple potential deprotonation sites, allowing a robust assessment of preferential ionization mechanisms. The computational methodology and basis set employed in this research are widely used in quantum chemical applications and have been considered suitable for providing consistent and comparable results with experimental measurements [21,22]. Moreover, these approaches have been successfully applied in similar studies involving lichen metabolites and other organic molecules, supporting the reliability and relevance of the present analysis [7].

## 4. Conclusions

In summary, this study represents the first comprehensive metabolomic characterization of the lichen *Hypotrachyna enderythraea* (Zahlbr.) Hale, identifying sixteen metabolites via HPLC–Orbitrap-ESI-MS/MS. The chemical profile includes a wide variety of compounds, such us chromones, dibenzofurans, lipids, depsides, aromatic derivatives such as quinone, and a terpene. Notably, *H. enderythraea* exhibited a distinct metabolic profile containing only usnic and salazinic acids with other species of the genus. Mass Spectrometry fragmentation patterns were found to be consistent with the even-electron rule, while DFT calculations on representative polyprotic metabolites elucidated that the deprotonation of specific hydroxyl or carboxyl groups contributes to the enhanced stability of their corresponding anions.

These results establish *H. enderythraea* as a significant species for further exploration of the genus *Hypotrachyna*. The detection of exclusive metabolites with established ecological and pharmacological roles—such as usnic, salazinic, lobaric, lepraric, and solorinic acids, along with pulvinic acid derivatives—represents an important finding with the aim of considering these metabolites as potential UV protective and antimicrobial agents. Therefore, these findings support further studies on the specific extractions of these secondary metabolites and evaluate their biological activities from diverse extract types. In addition, future investigations should isolate and quantify the most abundant metabolites in this species, as well as expand in silico analyses of the identified compounds with the aim of predicting their potential pharmacological activity. These methods will improve the chemical and pharmacological knowledge of this lichen species and offer deeper mechanistic insights into bioactivity.

## Figures and Tables

**Table 2:** Computational results at the B3LYP/6-311++G(d,p) level of theory for representative secondary polyprotic metabolites.

Secondary Metabolite	*G ^a^* (Hartree)	*H ^b^* (Hartree)	∆*G* = *GA^c,e^* (kcal/mol)	∆*H*_acid_ *^d,e^* (kcal/mol)
Salazinic acid **(2)**	−1445,5594	−1445,4824		
[**2**-H]^−^ deprot 14-O	−1445,0431	−1444,9649	317.7	326.2
[**2**-H]^−^ deprot 30-O	−1445,0623	−1444,9859	305.7	313.0
[**2**-H]^−^ deprot 35-O	−1445,0623	−1444,9859	305.7	313.0
[**2**-H]^−^ deprot 37-O	−1445,0389	−1444,9607	320.3	328.8
Coumarinic acid **(3)**	−573,5042	−573,4540		
[**3**-H]^−^ deprot 11-O	−572,9800	−572,9315	322.7	329.3
[**3**-H]^−^ deprot 18-O	−572,9675	−572,9186	330.5	337.4
6-Hydroxymethyl-eugenitin **(4)**	−840,6596	−840,6002		
[**4**-H]^−^ deprot 18-O	−840,1355	−840,0758	328.9	329.1
Lepraric acid **(7)**	−1298,6448	−1298,5582		
[**7**-H]^−^ deprot 18-O	−1298,1209	−1298,0321	322.6	330.5
[**7**-H]^−^ deprot 43-O	−1298,1206	−1298,0346	322.7	330.0
Lepraric acid **(7**_IS_**) *^f^***	−1298,6435	−1298,5565		
[**7**-H]^−^ deprot 18-O	−1298,1195	−1298,0315	322.5	330.9
[**7**-H]^−^ deprot 43-O	−1298,1206	−1298,0346	321.8	329.0

*^a^* ***G*** = Gibbs energy at 298 K. ***^b^ H*** = enthalpy at 298 K. ***^c^ GA*** = acidity in the gas phase. ***^d^* ∆*H*_acid_** = deprotonation enthalpy in the gas phase. ***^e^ GA*** and **∆*H*_acid_** of a protic acid AH are defined as Gibbs energy and enthalpy changes in the deprotonation reaction, AH (g) → A^−^(g) + H^+^(g) [66]. ***^f^*** Isomer of **7**.

## Data Availability

Data are contained within the manuscript.

## Supplementary Materials

The following supporting information can be downloaded at: https://www.mdpi.com/article/10.3390/molecules31060954/s1, Figure S1: Chromatogram of *Hypotrachyna enderythraea* (Zahlbr.) Hale on positive mode; Figure S2: MS^2^ spectra of the [M−H]^−^ anion assigned to Azelaic acid (**1**); Figure S3: MS^2^ spectra of the [M−H]^−^ anion assigned to Ethylhaematommate (**6**); Figure S4: MS^2^ spectra of the [M−H]^−^ anion assigned to Lobaric acid (**9**); Figure S5: MS^2^ spectra of the [M-H]^-^ anion assigned to Pseudoplacodiolic acid (**10**); Figure S6: (A) MS^2^ spectra of the [M-H]^-^ anion and (B) MS^2^ spectra of [M+H]^+^ cation assigned to Usnic acid (**11**); Figure S7: (A) MS^2^ spectra of the [M-H]^-^ anion and (B) MS^2^ spectra of [M+H]^+^ cation assigned to Isousnic acid (**12**); Figure S8: MS^2^ spectra of the [M-H]^-^ anion assigned to Palmitic acid (**13**); Figure S9: MS^2^ spectra of the [M-H]^-^ anion assigned to Stearic acid (**14**); Figure S10: MS^2^ spectra of the [M-H]^-^ anion assigned to 9-Methyl 8-O-methylpannarate (**15**); Figure S11: MS^2^ spectra of the [M-H]^-^ anion assigned to Portentol (**16**); Table S1: Identification of (R)-Usnic acid and Isousnic acid in the methanol:acetone (1:1, *v*/*v*) extract from *H. enderythraea* (Zahlbr.) Hale by HPLC-Orbitrap ESI-MS/MS in positive ion mode.

## Abbreviations

The following abbreviations are used in this manuscript:

HPLC	High-Performance Liquid Chromatography
ESI	Electrospray Ionization
MS/MS	Tandem Mass Spectrometry
HPLC-Orbitrap ESI MS/MS	High-Performance Liquid Chromatography coupled to Orbitrap Electrospray Ionization tandem Mass Spectrometry
[M+H]^+^	Protonated Molecular Ion (positive ion mode)
[M−H]^−^	Deprotonated Molecular Ion (negative ion mode)
RT	Retention Time
DFT	Density Functional Theory
MeOH	Methanol
Acet	Acetone
Hypotrachyna end.	*Hypotrachyna enderythraea* (Zahlbr.) Hale
