# Supplementary material for: Identification of Secondary Metabolites from the Lichen Hypotrachyna enderythraea (Zahlbr.) Hale by HPLC-ESI-MS/MS
Source: Molecules. 2026 Mar 12;31(6):954. doi: 10.3390/molecules31060954 (PMC13029706; doi:10.3390/molecules31060954)
Supplement: Supplementary file 1 [file molecules-31-00954-s001.zip › molecules-4124248-supplementary.pdf]

SUPPORTING INFORMATION

Associated with the paper

Identification of Secondary Metabolites from the Lichen *Hypotrachyna enderythraea* (Zahlbr.) Hale by HPLC-ESI-MS/MS

Fernando Carrasco [1,2,*], Wilfredo Hernández [1], Nino Castro [2], Nelly Sivipaucar [3], Bruno Bongiorno [3], Oscar Chupayo [3], Cesar Raposo [4], Lúcia A. Silva [5], Jesus M. Rodilla [5], Eduardo Carrasco [6] and Juan. Z. Dávalos [7]

[1] Facultad de Ingeniería, Universidad de Lima, Av. Javier Prado Este 4600, Lima 15023, Peru; rhenand@ulima.edu.pe

[2] Departamento Académico de Química Orgánica, Escuela de Química, Facultad de Química e Ingeniería Química, Universidad Nacional Mayor de San Marcos, Calle German Amezaga 375, Lima 15081, Peru; ocastrom@unmsm.edu.pe

[3] Facultad de Ciencias Naturales y Matemática, Universidad Nacional Federico Villarreal, Jr. Río Chepen s/n, El Agustino; Lima 15007, Peru; sivipaucarnellyelena@gmail.com (N.S.); brunobongiornoe@gmail.com (B.B.); oscarchupayo@gmail.com (O.C.)

[4] Mass Spectrometry Service, NUCLEUS, University of Salamanca, 37008 Salamanca, Spain; raposo@usal.es

[5] Faculdade de Ciencias, Departamento de Quıímica and Fiber Materials and Environmental Technologies (FibEnTech-UBI), Universidade da Beira Interior, R. Marquês de D'Ávila e Bolama, 6201-001 Covilhã, Portugal; mlas@ubi.pt (L.A.S.); rodilla@ubi.pt (J.M.R.)

[6] Facultad de Ciencias Física, Universidad Nacional Mayor de San Marcos, Lima 15081, Peru; ecarrascoso@unmsm.edu.pe

[7] Instituto de Química-Física "Blas Cabrera", CSIC, Serrano 119, 28006 Madrid, Spain; jdavalos@iqf.csic.es

* Correspondence: fccarras@ulima.edu.pe

Table S1. Identification of (R)-Usnic acid and Isousnic acid in the methanol:acetone (1:1, v/v) extract from *H. enderythraea* (Zahlbr.) Hale by HPLC-Orbitrap ESI-MS/MS in positive ion mode.

Peak	Compounds	$[M+H]^+$	Ret. time (min.)	Theoretical (m/z)	Experimental (m/z)	Accuracy (ppm)	Fragmentation Pattern	Type	Ref.
**11**	(R)-Usnic acid	$C_{18}H_{17}O_7$	10.6	345.0967	345.0965	-0.6	327.086; 261.0756; 233.0806; 215.0702	**DBF**	**[7, 22, 41]**
**12**	Isousnic acid	$C_{18}H_{17}O_7$	11.04	345.0967	345.0966	-0.3	327.0861; 309.0757; 285.0757; 233.0806.	**DBF**	**[39, 44]**

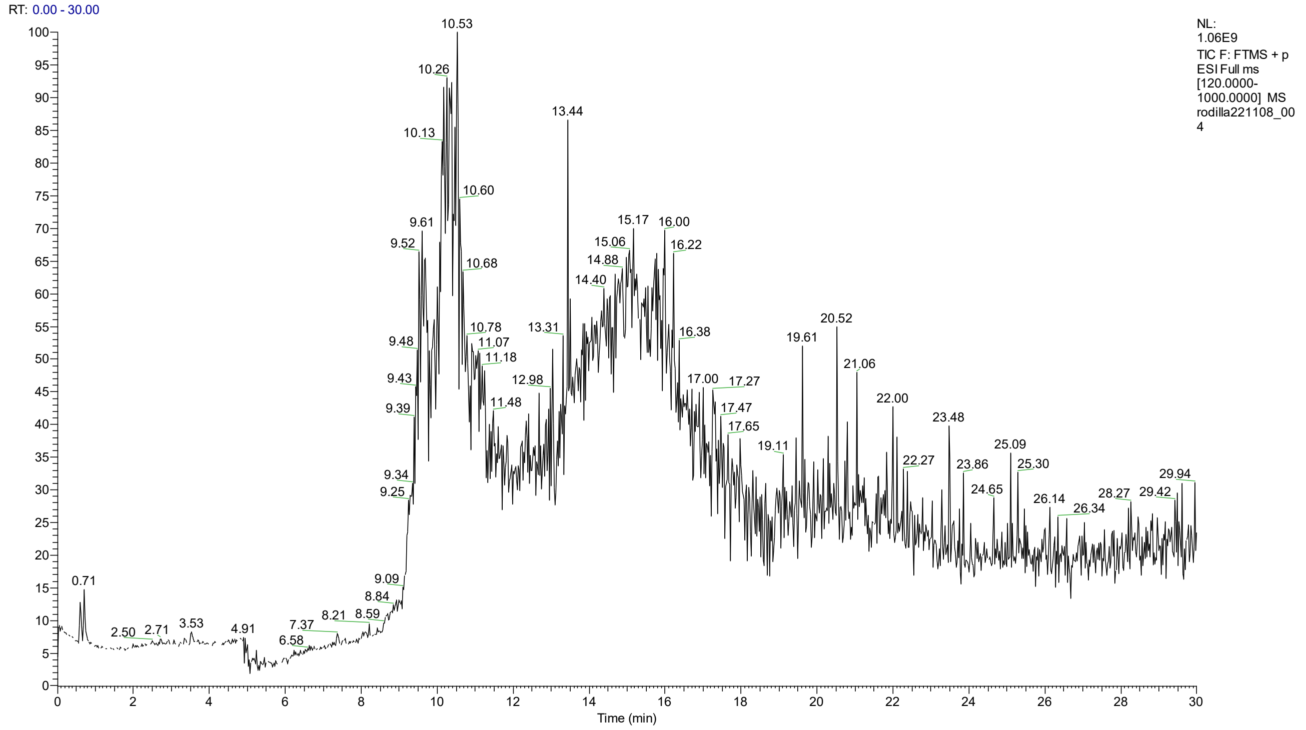

Figure S1. Chromatogram of *Hypotrachyna enderythraea* (Zahlbr.) Hale on positive mode

**1**	Azelaic acid	4.23	$C_9H_{16}O_4$	188.105		187.097	169.0864; 125.0962; 123.0808; 97.0649

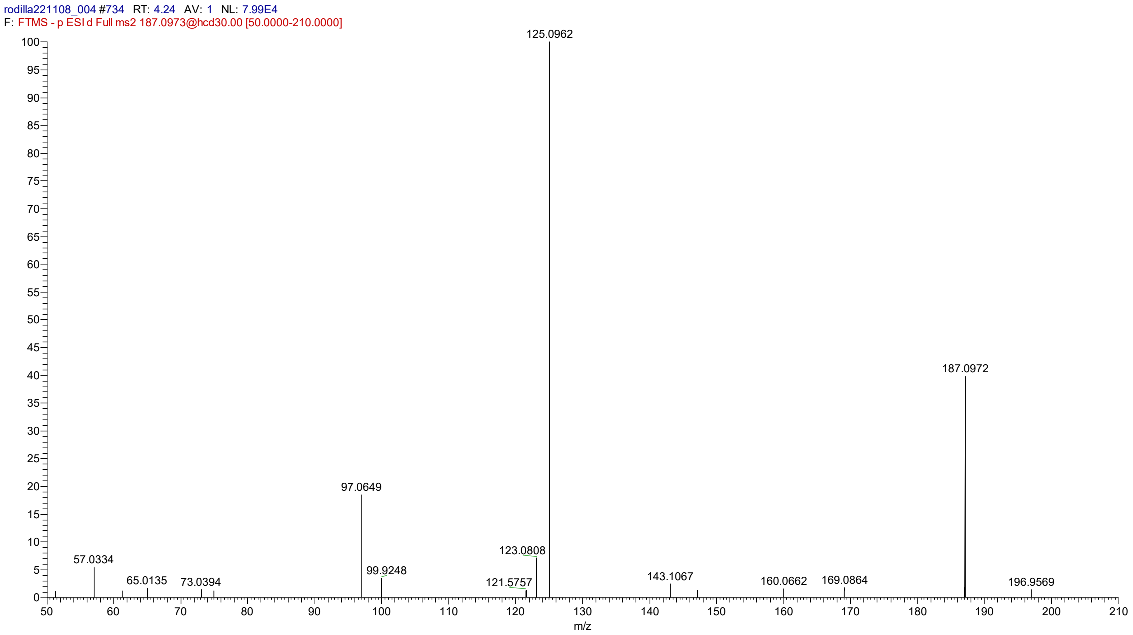

Figure S2. MS² spectra of the [M-H]- anion assigned to Azelaic acid (**1**)

**6**	Ethyl haematommate	7.56	$C_{11}H_{12}O_5$	224.0689		223.061	207.0297; 181.05; 163.0391; 83.0127

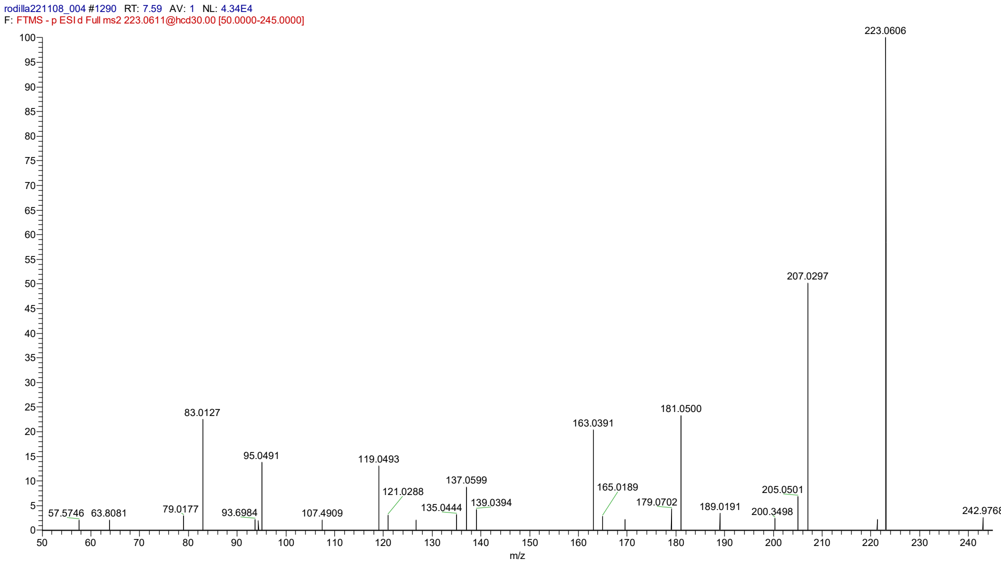

Figure S3. MS² spectra of the $[M-H]^-$ anion assigned to Ethylhaematommate (**6**)

**9**	Lobaric acid	9.24	$C_{25}H_{28}O_8$	456.1794		455.172	367.1911; 309.1143; 295.0997; 281.0828

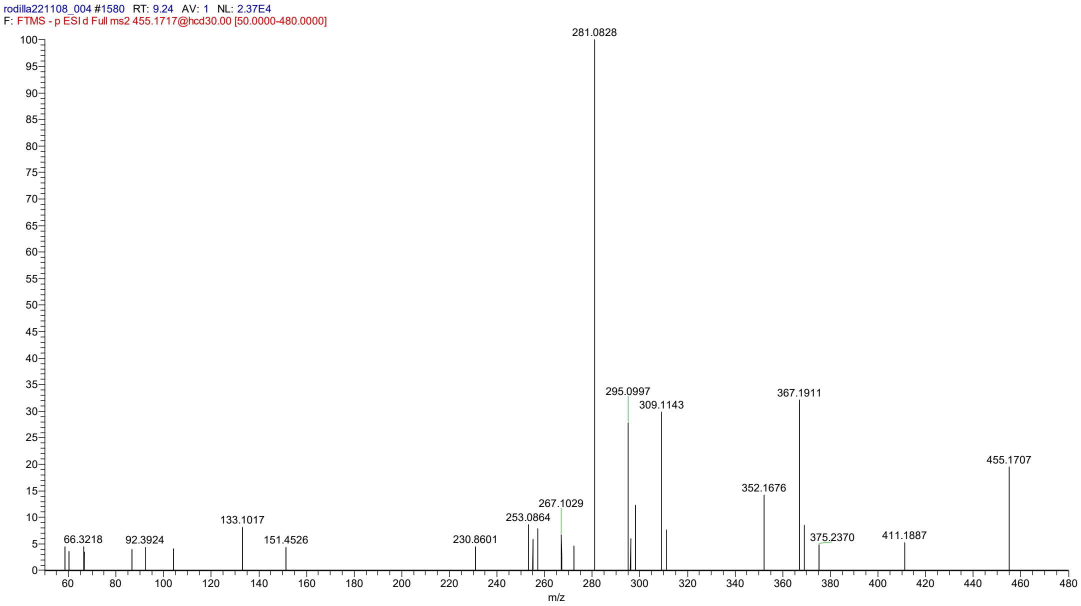

Figure S4. MS² spectra of the [M-H]⁻ anion assigned to Lobaric acid (**9**)

**10**	Pseudoplacodiolic acid	9.69	$C_{19}H_{20}O_8$	376.1166		375.109	299.0393; 255.0666; 231.0663; 83.0127

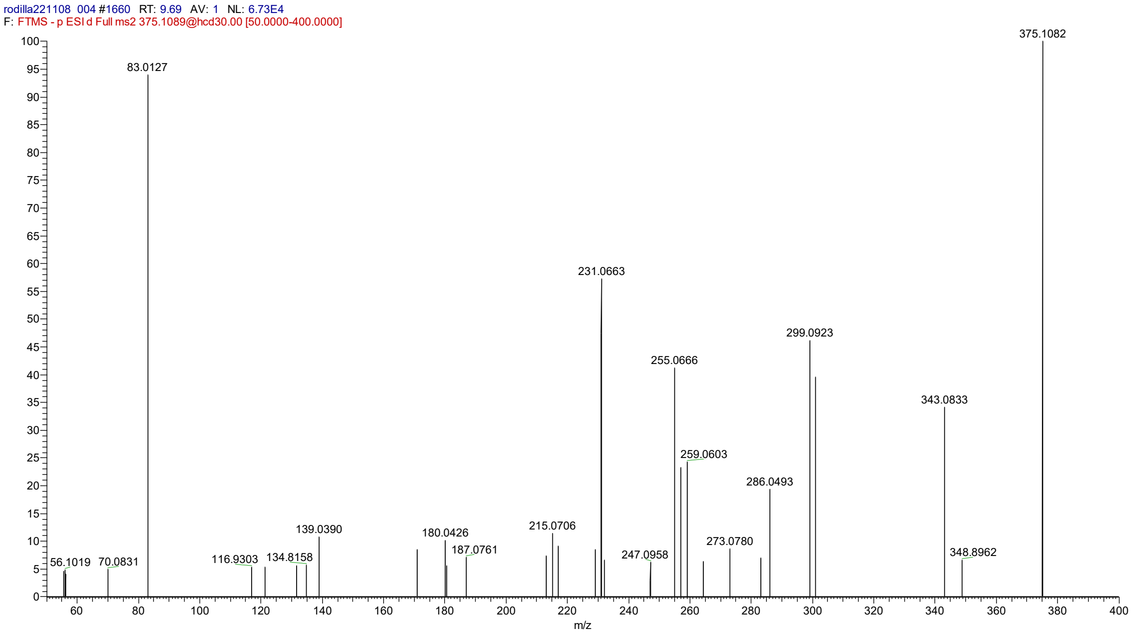

Figure S5. MS² spectra of the [M-H]⁻ anion assigned to Pseudoplacodiolic acid (**10**)

**11**	(R)-Usnic acid	10.6	$C_{18}H_{16}O_7$	344.0887	345.097	343.082	328.0594; 259.0616; 231.0663; 83.0128. 327.086; 261.0756; 233.0806; 215.0702.

Negative ionization

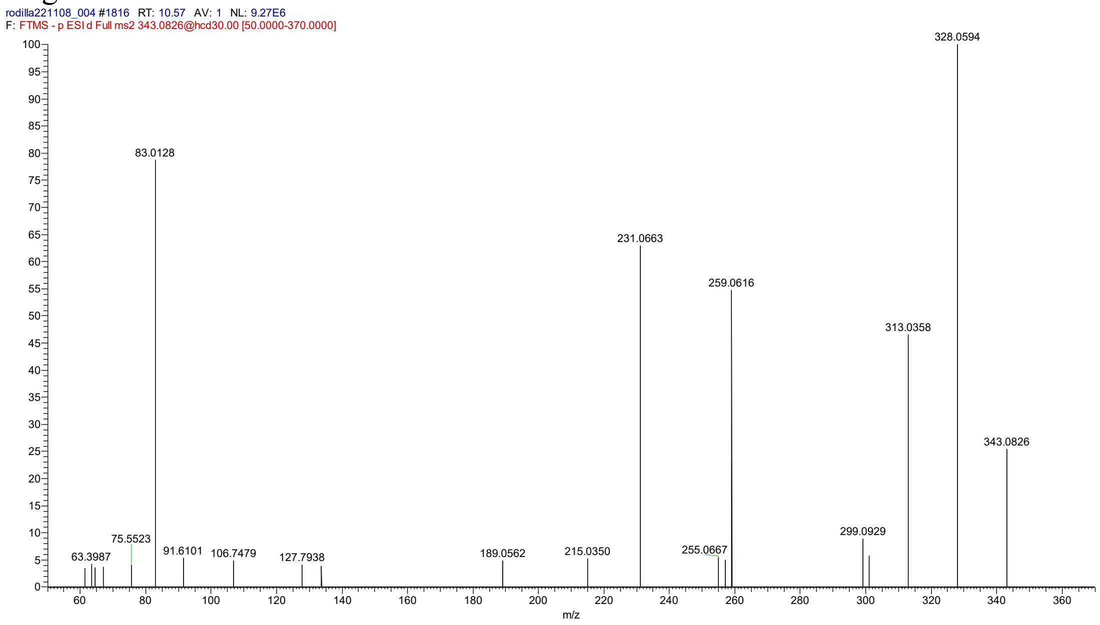

(A)

Positive ionization

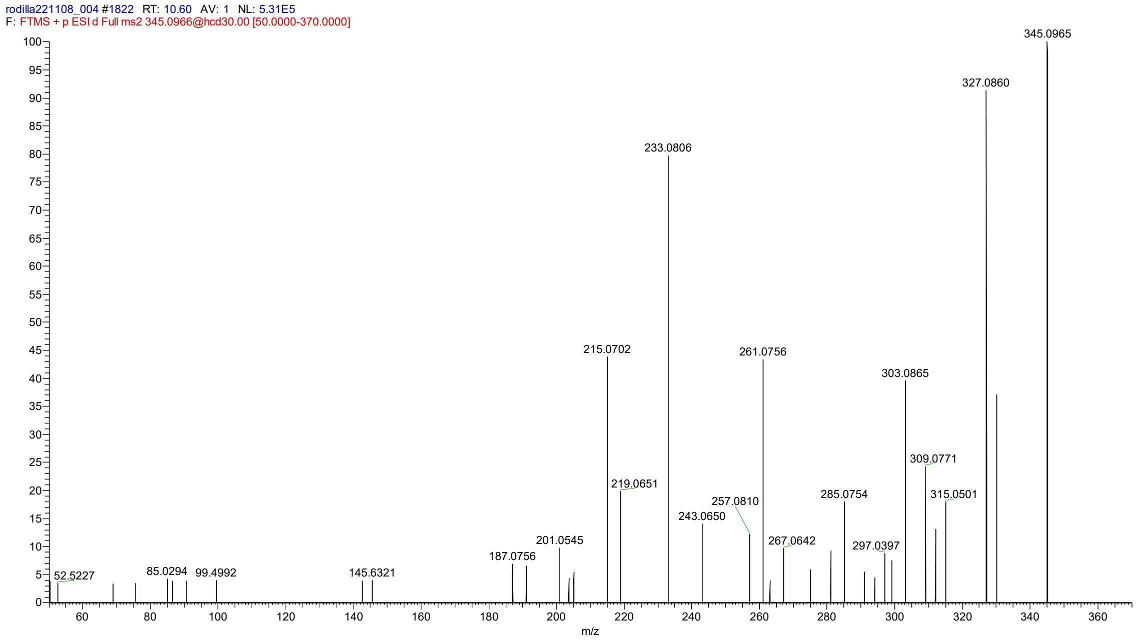

(B)

Figure S6. (A) MS² spectra of the [M-H]⁻ anion and (B) MS² spectra of [M+H]⁺ cation assigned to Usnic acid (**11**)

**12**	Isousnic acid	11.04	$C_{18}H_{16}O_7$	344.0888	345.097	343.082	328.0592; 313.0356; 231.0663; 83.0128<br>327.0861; 309.0757; 285.0757; 233.0806.

Negative ionization

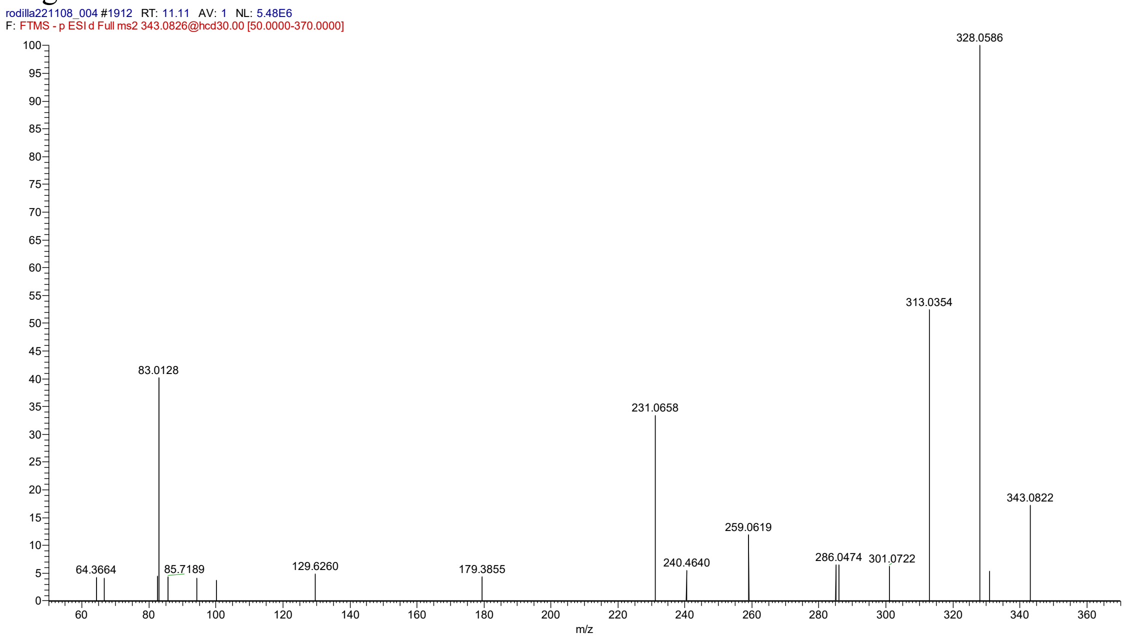

(A)

Positive ionization

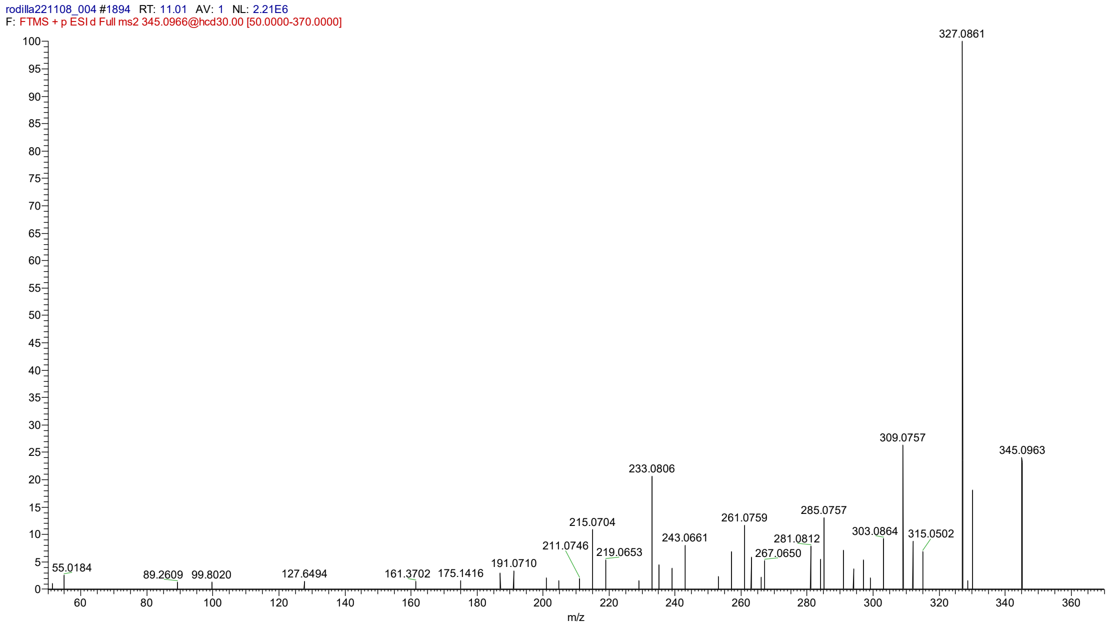

(B)

Figure S7. (A) MS² spectra of the [M-H]⁻ anion and (B) MS² spectra of [M+H]⁺ cation assigned to Isousnic acid (**12**)

**13**	Palmitic acid	13.47	$C_{16}H_{32}O_2$	256.241		255.233	177.689; 116.9272; 97.5035

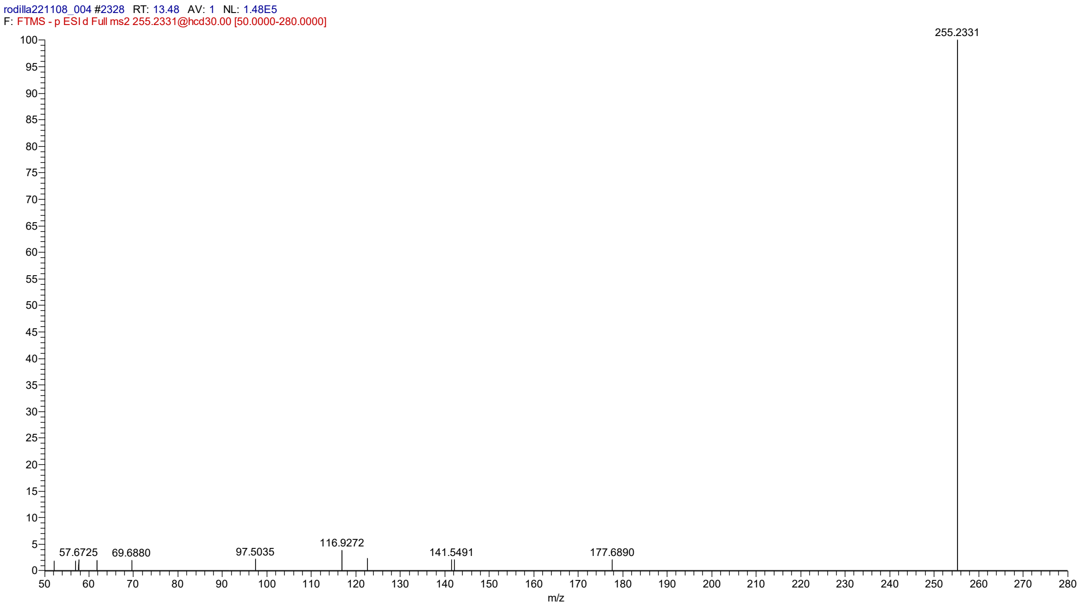

Figure S8. MS² spectra of the [M-H]⁻ anion assigned to Palmitic acid (**13**)

**14**	Stearic acid	15.18	$C_{18}H_{36}O_2$	284.2724		283.265	259.6608; 200.7311; 138.0842; 86.0265

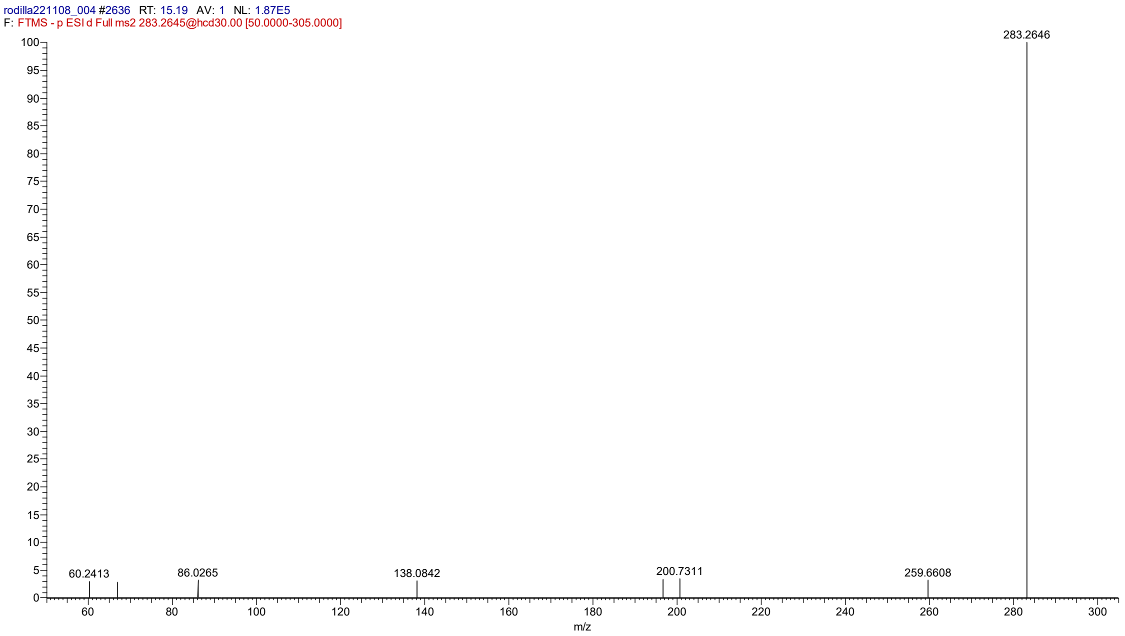

Figure S9. MS² spectra of the [M-H]⁻ anion assigned to Stearic acid (**14**)

**15**	9-Methyl 8-O-methylpannarate	15.2	$C_{18}H_{16}O_7$	344.0905		343.083	328.0589; 313.0358; 231.0659; 83.0127

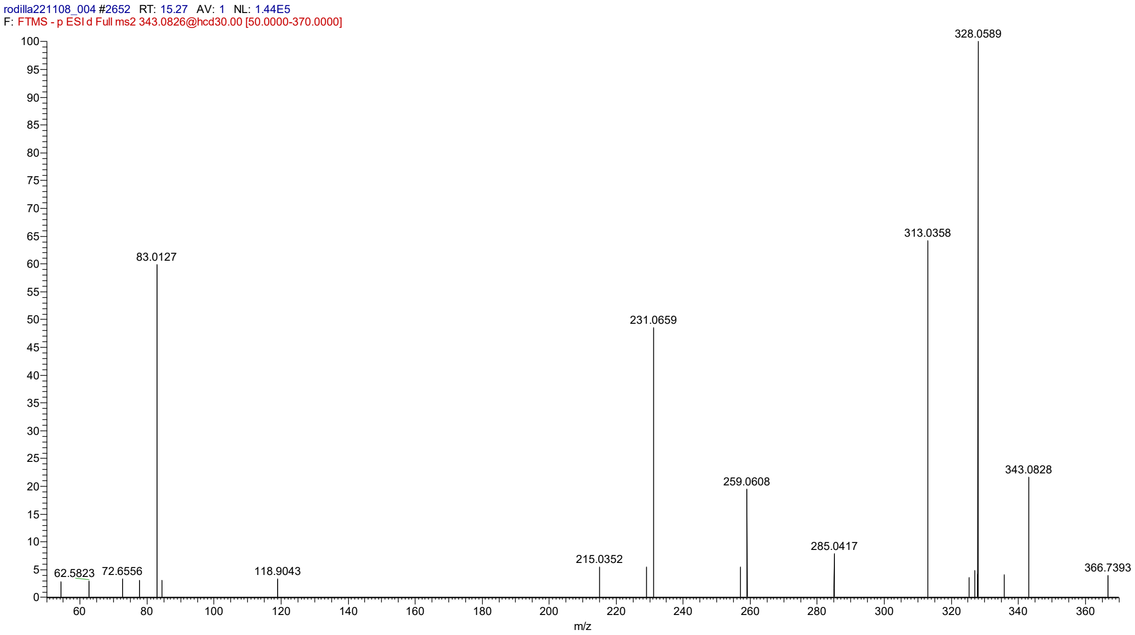

Figure S10. MS² spectra of the [M-H]⁻ anion assigned to 9-Methyl 8-O-methylpannarate (**15**)

**16**	Portentol	21.47	$C_{17}H_{26}O_5$	310.1822		309.174	235.8537; 122.9746; 96.959; 79.9564

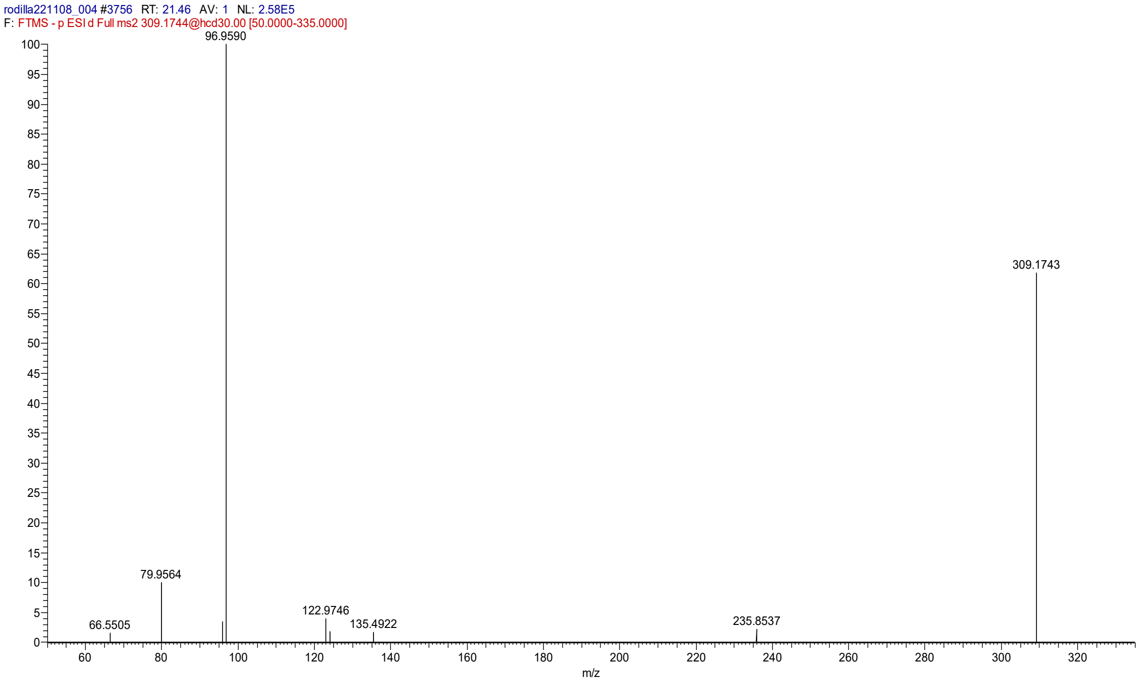

Figure S11. MS² spectra of the [M-H]⁻ anion assigned to Portentol (**16**)
